# Prognostic meaning of right ventricular function and output reserve in patients with systemic sclerosis

**DOI:** 10.1186/s13075-022-02863-1

**Published:** 2022-07-21

**Authors:** Panagiota Xanthouli, Julia Miazgowski, Nicola Benjamin, Ojan Gordjani, Benjamin Egenlauf, Satenik Harutyunova, Rebekka Seeger, Alberto M. Marra, Norbert Blank, Hanns-Martin Lorenz, Ekkehard Grünig, Christina A. Eichstaedt

**Affiliations:** 1 Centre for Pulmonary Hypertension, Thoraxklinik Heidelberg gGmbH at Heidelberg University Hospital, Heidelberg, Germany; 2 Translational Lung Research Centre Heidelberg (TLRC), Member of the German Centre for Lung Research (DZL), Heidelberg, Germany; 3 Department of Pneumology and Critical Care Medicine, Thoraxklinik Heidelberg gGmbH at Heidelberg University Hospital, Heidelberg, Germany; 4 Department of Internal Medicine V: Hematology, Oncology and Rheumatology, University Hospital Heidelberg, Heidelberg, Germany; 5 Department of Translational Medical Sciences, “Federico II” University and School of Medicine, Naples, Italy; 6 Laboratory for Molecular Genetic Diagnostics, Institute of Human Genetics, Heidelberg University, Heidelberg, Germany

**Keywords:** Systemic sclerosis, Right ventricular reserve, Pulmonary hypertension, Screening, Echocardiography, Right heart catheterisation

## Abstract

**Background:**

The objective of this study was to investigate the prognostic impact of right ventricular (RV) function at rest and during exercise in patients with systemic sclerosis (SSc) presenting for a screening for pulmonary hypertension (PH).

**Methods:**

In this study, data from SSc patients who underwent routinely performed examinations for PH screening including echocardiography and right heart catheterization at rest and during exercise were analysed. Uni- and multivariable analyses were performed to identify prognostic parameters.

**Results:**

Out of 280 SSc patients screened for PH, 225 were included in the analysis (81.3% female, mean age 58.1±13.0 years, 68% limited cutaneous SSc, WHO-FC II–III 74%, 24 manifest PH). During the observation period of 3.2±2.7 (median 2.6) years 35 patients died. Tricuspid annular plane systolic excursion (TAPSE) at rest <18 mm (*p*=0.001), RV output reserve as increase of cardiac index (CI) during exercise <2 l/min (*p*<0.0001), RV pulmonary vascular reserve (Δ mean pulmonary artery pressure/Δ cardiac output) ≥3 mmHg/l/min (*p*<0.0001), peak CI <5.5 l/min/m^2^ (*p*=0.001), pulmonary arterial compliance <2 ml/mmHg (*p*=0.002), TAPSE/systolic pulmonary arterial pressure (sPAP) ratio ≤0.6 ml/mmHg (*p*<0.0001) and echocardiographic qualitative RV function at rest (*p*<0.0001) significantly predicted worse survival. In the multivariable analysis TAPSE/sPAP ratio and diffusion capacity for carbon monoxide ≤65% were identified as independent prognostic predictors and had 75% sensitivity and 69% specificity to predict future development of pulmonary vascular disease (PVD) during follow-up.

**Conclusions:**

This study demonstrates that assessment of RV function at rest and during exercise may provide crucial information to identify SSc patients who are at a high risk of poor outcome and for the development of PH and/or PVD.

## Introduction

Systemic sclerosis (SSc) is a rare autoimmune connective tissue disorder, affecting skin and different internal organs [1]. In about 15–25% of symptomatic and 8-12% of asymptomatic SSc patients the disease manifests in a concomitant pulmonary hypertension (PH) [2, 3]. In absence of comorbidities, such as heart disease or lung fibrosis, the disease can be classified as SSc-associated pulmonary arterial hypertension (SSc-APAH). At pulmonary arterial hypertension (PAH) diagnosis, >85% of patients with SSc are already in advanced stages of the disease (World Health Organization functional classes (WHO-FC) III and IV) [4]. Untreated patients present with a markedly reduced 3-year survival of 56%, compared with 91% in SSc patients without PAH [5]. Another study showed a short median survival in SSc-APAH patients of only four years despite an advanced PAH therapy [6]. Therefore, an early diagnosis of pulmonary vascular disease (PVD) is essential and screening programmes in SSc patients are recommended [7–10]. The DETECT study developed an evidence-based algorithm for the early detection of APAH in SSc patients [11] using clinical and echocardiographic data to determine patient referral for right heart catheterisation (RHC) to confirm PH/PAH diagnosis. At the time of the DETECT algorithm development, pre-capillary PH was haemodynamically defined as mean pulmonary arterial pressure (mPAP) ≥25 mmHg and pulmonary capillary wedge pressure (PAWP) ≤15 mmHg, measured by RHC [7]. In the following ESC/ERS guidelines 2015, pulmonary vascular resistance (PVR) >3 Wood Units (WU) was included in the haemodynamic definition of PAH [10].

Using the DETECT algorithm or RHC at rest for screening, the majority of the newly diagnosed pre-capillary PH patients presented with only slightly elevated mPAP, normal or near-normal mean cardiac output (CO) at rest, slightly elevated right atrial size and a PVR value <3 WU [11–14].

During the 6^th^ World Symposium on PH, a new haemodynamic definition was suggested to enable early diagnosis, by lowering the mPAP threshold to >20 mmHg and to include PVR ≥3 WU as part of the haemodynamic definition for all forms of pre-capillary PH [15–17]. More recently, studies provided evidence that even a lower threshold of ≥2 WU indicates abnormal PVR [18–20].

Patients with mildly elevated mPAP (who would meet the new diagnostic criteria for precapillary PH) showed already a reduced right ventricular (RV) output reserve (defined as reduced cardiac index (CI)-increase during exercise) and reduced pulmonary arterial compliance (PAC) suggestive for an early PVD [13].

It remains unclear, if RV function during exercise (RV output reserve) is of prognostic relevance for these patients. Furthermore, various parameters reflecting right heart size and RV function have been shown to be prognostically important in patients with PAH [21–25]. It is unclear, whether these parameters may also be used for estimation of prognosis in SSc patients (with or without PH) assessed for PH screening.

Therefore, the aim of this study was to investigate the prognostic impact of RV function and output reserve in patients with SSc presenting for PH screening and to compare them to established prognostic predictors. Furthermore, we aimed to assess, if changes in RV function at rest or during exercise are predictive for the development of PH during follow-up in this SSc cohort.

## Methods

### Study population and design

In this retrospective, monocentric cohort study adult patients with SSc who were assessed by both, non-invasive clinical procedures such as echocardiography and by RHC at rest and during exercise, at the PH-Centre in the Thoraxklinik Heidelberg gGmbH at Heidelberg University Hospital were enrolled. All retrospectively analysed patients were already diagnosed with SSc fulfilling the classification criteria of the American College of Rheumatology/European League against Rheumatism [26] and were categorised as patients with diffuse cutaneous SSc (dcSSc) or limited cutaneous SSc (lcSSc) [27]. Referring specialists were rheumatologists, cardiologists, pulmonologists and general practitioners.

Individuals were excluded, if they had no screening for PH, had rheumatic diseases other than SSc or were unable to give informed consent. Patients with missing haemodynamic assessment during exercise were excluded from data analysis. A part of this cohort has already been analysed and published before [11, 13, 18, 28].

All patients underwent a detailed screening for PH including medical history and physical examination, WHO-FC assessment, 6-min walking distance (6MWD), electrocardiogram, body plethysmography, diffusion capacity measurement of the lung for carbon monoxide (DLCO), blood gas analysis (capillary), computed tomography scan of the lung or other imaging, determination of laboratory parameters (especially N-terminal pro-brain natriuretic peptide (NT-proBNP) level), echocardiography at rest and during exercise, as well as RHC at rest and during exercise, standardised according to the current recommendations, as described before [29–31]. All data were pseudonymized. The study was approved by the ethics committee of the Medical Faculty of Heidelberg University (internal number S-305/2021). The study complied with the Declaration of Helsinki in its current version.

### Parameters of right ventricular function and reserve

Parameters reflecting RV function and output reserve were obtained from echocardiographic assessments [21, 32] and by right heart catheterisation at rest and during exercise [31, 33–37]. *Echocardiographic parameters* included right ventricular fractional area change (RV-FAC), tissue Doppler imaging tissue velocity (TDI TV s), qualitative right ventricular function, tricuspid annular plane systolic excursion (TAPSE) and TAPSE/systolic pulmonary arterial pressure ratio (sPAP) assessed by echocardiography. Qualitative function assessed during echocardiography was expressed as normal, mild, moderate or severe impairment. RV-FAC is a global measure of RV systolic function and was calculated as the difference between end-diastolic and end-systolic area, divided by the end-diastolic area. Furthermore, the velocity of the tricuspid valve characterises the pulmonary vascular flow.

*Parameters determined by RHC* included CI at rest and during exercise, mPAP, stroke volume, PVR, PAC, and RV pulmonary vascular reserve (mPAP increase/CO increase = ΔmPAP/ΔCO) and peak CI. CI at rest was used to describe RV function at rest, while an increase of CI defines RV output reserve. PAC was calculated as a specific measure to define abnormal vascular elasticity and was calculated as stroke volume/pulse pressure (stroke volume = CO/heart rate; pulse pressure = systolic pulmonary arterial pressure/diastolic pulmonary arterial pressure) [38]. All parameters were assessed according to current standards [29–31] and as described previously [18, 28].

Known thresholds for the above-listed parameters include TAPSE <18 mm, having already shown prognostic significance [39, 40], TAPSE/sPAP ratio ≤0.6 mm/mmHg [41] and RV pulmonary vascular reserve of ≥3 ml/min/mmHg [42], which has been used as the definition of exercise pulmonary hypertension [43].

### Known prognostic predictors in SSc

Parameters which have already been identified as prognostic predictors in SSc were used for comparison with parameters of RV function and reserve. Known prognostic predictors included age ≥60 years at baseline, DLCO ≤65% predicted [44–46], presence of pulmonary fibrosis [46] and PVR ≥2 WU [28].

### Statistical methods

Statistical analyses were conducted by a medical statistician (NB). Data are described as mean ± standard deviation (SD) with 95% confidence interval of the mean. Frequency data is given as number and percent.

Variables were evaluated by uni- and multivariable analysis to identify their impact on survival. Survival time was estimated from baseline (time of first screening assessment) until the end of follow-up in this study. Death was defined as death due to any cause. Cox regression analysis was used for survival analysis of continuous parameters. Parameters with significant prediction of survival in Cox regression analysis were dichotomized by receiver operating characteristic (ROC), or by threshold values from the literature. Kaplan-Meier analysis was performed with categorial parameters. Multivariable analysis was performed including significant predictors of RV function or output reserve and known prognostic predictors of SSc. Qualitative RV function was dichotomised as normal function vs. any impairment (including mild, moderate and severe impairment) due to insufficient sample size within the categories.

A multivariable risk set was analysed regarding prognostic prediction by Kaplan-Meier analysis including combination of independent prognostic predictors. An age-corrected Cox regression of the multivariable risk set was performed as sensitivity analysis. Multivariable analysis was performed leaving out patients with cancer as cause of death as sensitivity analysis. Furthermore, the multivariable analysis was performed in a subset of SSc patients without any signs of PVD, i.e. patients with mPAP 21-24 mmHg and PVR ≥2 WU, patients with mPAP ≥25 mmHg were excluded from the analysis. Differences of clinical parameters between patients presenting with none or a single vs. two or more risk factors were analysed. Clinical parameters were compared with the two-sided Student’s *t*-test for independent random samples. Frequency data were analysed using the chi-square test.

*P*-values <0.05 were considered to indicate statistical significance. All analyses have been performed using IBM SPSS 27 (SPSS Statistics V.27, IBM Corporation, Somers, NY, USA).

## Results

### Baseline characteristics (Table 1)

**Fig. 1:**
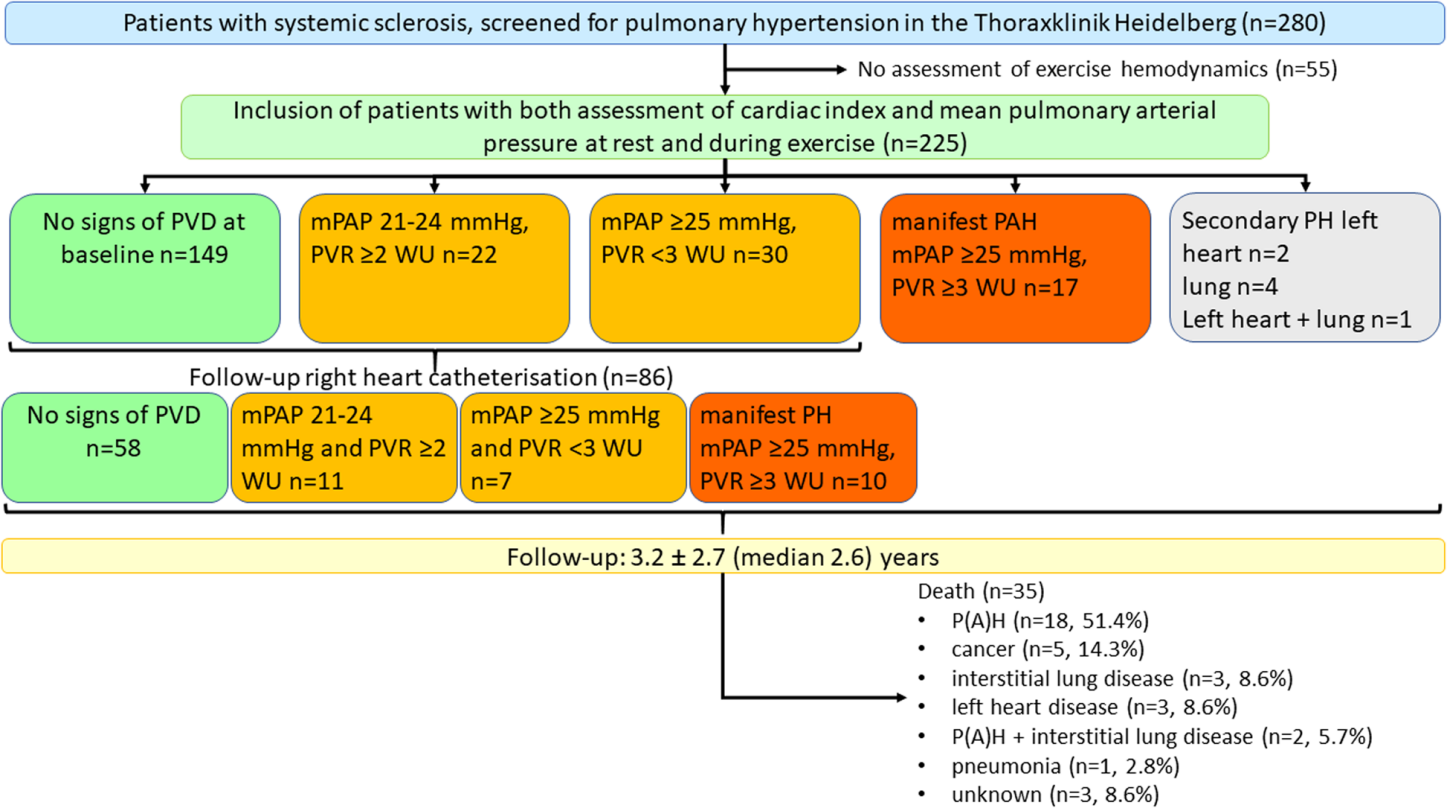
Study flow-chart. The graph provides information on patient-flow, baseline haemodynamics and follow-up. mPAP, mean pulmonary artery pressure; P(A)H, pulmonary (arterial) hypertension; PAWP, pulmonary arterial wedge pressure; PVD, pulmonary vascular disease; PVR, pulmonary vascular resistance

A total of 280 patients with SSc were screened for PH at the Centre for Pulmonary Hypertension at the Thoraxklinik Heidelberg between 2008 and 2020. Fifty-five patients were excluded from the study due to missing haemodynamic assessments during exercise (Fig. 1). Thus, the final dataset consisted of 225 patients (mean age 58.14 ± 12.95 years, 81.3% female, 67.6% lcSSc, 24.0% dcSSc) who had been assessed at baseline and during a follow-up time of 3.2±2.7 (median 2.6) years (Table 1).

**Table 1:** Characteristics of study cohort

	Whole cohort (***n***=225)				
	mean ± SD or ***n*** and (%)			95% confidence interval	n*
**Characteristics**					
Female sex, no [%]	183		81.3 %		
Age [years]	58.14	±	12.95	56.44 to 59.84	225
Height [cm]	166.13	±	08.63	165.00 to 167.26	225
Weight [kg]	70.85	±	15.80	68.78 to 72.93	225
Systolic blood pressure [mmHg]	133.00	±	21.14	130.19 to 135.82	219
Diastolic blood pressure [mmHg]	76.02	±	10.93	74.57 to 77.48	219
Heart rate at rest [/min]	75.63	±	12.36	73.46 to 77.80	127
**Type of systemic sclerosis**					225
Limited cutaneous SSc [%]	152		67.6%		
Diffuse cutaneous SSc [%]	54		24.0%		
Early SSc	19		8.4%		
**Duration SSc [days]**	3420.54	±	3325.72	2980.65 to 3860.42	222
**SSc characteristics**					
Modified Rodnan skin score	12	±	10	10 to 13	160
Digital ulcers	76		33.9%		224
**WHO-FC, no [%]**					
I	54		24.0%		54
II	108		48.0%		108
III	59		26.3%		53
IV	4		1.8%		4
**Lung function**					
Vital capacity max [l]	2.96	±	0.95	2.84 to 3.09	221
Forced expiratory volume in one second [l]	2.35	±	0.78	2.25 to 2.45	222
Total lung capacity [l]	5.05	±	1.29	4.88 to 5.22	221
Diffusion capacity of the lung for carbon monoxide SB [%]	57.50	±	18.27	55.02 to 59.98	211
Diffusion capacity of the lung for carbon monoxide/VA [%]	72.51	±	19.90	69.82 to 75.20	213
Mixed venous oxygen saturation [%]	72.13	±	12.93	69.36 to 74.90	86
**Laboratory**					
N-terminal pro-brain natriuretic peptide [ng/l]	374.31	±	718.49	274.12 to 474.49	200
C-reactive protein [mg/l]	6.95	±	9.42	5.7 to 8.19	222
Troponin T [μg/l]	125	±	11.62	8.45 to 12.57	125
Glomerular filtration rate [ml/min/1.73m^2^]	85.81	±	26.36	82.2 to 89.43	207
Creatinine [mg/dl]	0.88	±	0.88	0.76 to 1.0	222
**6-minute walking distance [meters]**	439.93	±	96.18	426.72 to 453.14	206
**Echocardiography**					
Right atrial area [cm^2^]	12.38	±	3.73	11.88 to 12.88	214
Right ventricular area [cm^2^]	14.81	±	3.66	14.33 to 15.30	218
Systolic pulmonary arterial pressure [mmHg]	32.10	±	13.43	30.30 to 33.90	216
Tricuspid annular plane systolic excursion [mm]	23.93	±	4.45	23.34 to 24.53	218
Right ventricular fractional area change [%]	0.71	±	0.32	0.63 to 0.78	69
Tissue Doppler imaging tissue velocity S	14	±	3	13 to 14	182
Increase of systolic pulmonary arterial pressure during exercise [mmHg]	25.78	±	10.65	23.80 to 27.76	114
**Right heart catheter**					
Mean pulmonary arterial pressure [mmHg]	20.41	±	8.43	19.31 to 21.52	225
Cardiac output [l/min]	5.39	±	1.42	5.20 to 5.57	225
Pulmonary arterial wedge pressure [mmHg]	9.39	±	4.18	8.84 to 9.94	225
Pulmonary vascular resistance [WU]	2.22	±	1.73	1.99 to 2.45	225
Pulmonary arterial compliance [ml/mmHg]	4.41	±	2.31	4.08 to 4.74	195
RV pulmonary vascular reserve [mmHg/l/min]	7.79	±	43.62	2.06 to 13.52	225
Cardiac index [l/min/m^2^]	3.08	±	0.78	2.98 to 3.18	225
Cardiac index increase during exercise [l/min/m^2^]	2.78	±	1.59	2.57 to 2.98	225
Peak cardiac index [l/min/m^2^]	5.86	±	1.69	5.63 to 6.08	225
**Right ventricular pump function**					221
Normal	199		90.0%		
Mild impairment	11		5.0%		
Moderate impairment	4		1.8%		
Severe impairment	7		3.2%		

*SB* single breath, *SSc* systemic sclerosis, *VA* alveolar volume, *WHO-FC* World Health Organization functional class

*In case of missing values, *n* is provided

Concomitant arterial hypertension was present in 77 patients (34.4%) and 90 patients (40%) presented with pulmonary fibrosis. In 76.1% of the patients, a functional impairment was described with WHO-FC ≥II (Table 1). Haemodynamic assessments generally showed normal mean values of right heart size and function (Table 1) [47]. In 22 patients, RV pump function was impaired at baseline (mild to severe impairment).

At baseline 149 of 225 patients (66.2%) had normal pulmonary haemodynamics at rest (Fig. 1). In 24 patients (10.6%) mPAP and PVR were increased, meeting the definition of manifest PH (Fig. 1). Out of these patients, 17 (7.6%) were classified as SSc-APAH, two presented with PH due to left heart disease, four with PH due to lung disease and one PH patient had both left heart and lung disease. In further 52 patients (23.1%), early signs of PVD were detected at baseline with mildly elevated mPAP 21-24 mmHg and PVR ≥2 WU at rest (*n*=22) or mPAP ≥25 mmHg and PVR ≤3 WU (*n*=30).

### Survival in SSc patients and prognostic predictors

During an observation period of 3.2±2.7 (median 2.6) years 35 patients died. Reasons for death were P(A)H (*n*=18, 51.4%), cancer (*n*=5, 14.3%), pulmonary fibrosis (*n*=3, 8.6%), left heart disease (*n*=3, 8.6%), P(A)H and pulmonary fibrosis (*n*=2, 5.7%), pneumonia (*n*=1, 2.8%) and unknown cause of death (*n*=3, 8.6%).

Significant predictors for survival in the *univariable Cox regression analysis* were TAPSE, TAPSE/sPAP, PAC, CI increase, RV pulmonary vascular reserve, peak CI and qualitative RV function (Table 2). The threshold for impaired RV function from the literature was <18 mm for TAPSE. RV pulmonary vascular reserve ≥3 mmHg/(l/min) was used as threshold for mPAP / CO slope, indicating exercise PH. For parameters with no established thresholds, ROC analysis was performed, leading to thresholds of <2 ml/mmHg for PAC, <5.5 l/min/m^2^ for peak CI, <2 l/min/m^2^ for CI increase indicating impaired RV function or reserve.

**Table 2:** Univariable survival analysis

	**Univariable analysis**			
**Cox Regression analysis**	***p*-value**	**Exp(B)**		
Tricuspid annular plane systolic excursion (TAPSE)	0.003	0.862		
Fractional area change	0.918	0.919		
TDI TV s	0.777	0.973		
Pulmonary arterial compliance	0.001	0.759		
Cardiac index at rest	0.314	0.707		
Cardiac index increase	0.001	0.654		
RV pulmonary vascular reserve (mPAP increase/CO increase)	0.049	1.006		
Cardiac index peak	0.001	0.672		
Systolic pulmonary arterial pressure (sPAP)	0.217	1.027		
TAPSE/sPAP	<0.0001	0.063		
Right ventricular function - normal vs. any impairment	<0.0001	0.205		
**Kaplan-Meier analysis (categorial parameters)**			**Multivariable analysis stepwise forward selection**	
**RV function and reserve**			***p*-value**	**Exp(B)**
Tricuspid annular plane systolic excursion <18 mm	0.001			
Right ventricular function - normal vs. any impairment	<0.0001			
Pulmonary arterial compliance <2 ml/mmHg	0.002			
Cardiac index increase <2 l/min/m^2^	<0.0001			
RV pulmonary vascular reserve ≥3 mmHg/(l/min) (literature)	<0.0001			
Cardiac index peak <5.5 l/min	0.001			
TAPSE/sPAP ≤0.6 mm/mmHg	<0.0001		<0.0001	0.201
**Known prognostic predictors**				
Age ≥60 years	0.004			
DLCO ≤65% predicted	<0.0001		0.003	0.297
Sex	0.554			
Type of systemic sclerosis	0.497			
Pulmonary fibrosis	0.034			
Pulmonary vascular resistance ≥2 Wood Units	0.002			
**Combination of independent prognostic predictors (Kaplan-Meier)**				
DLCO ≤65% and TAPSE/sPAP	<0.0001			

*TDI* tissue Doppler imaging, *TV* tissue velocity, *RV* right ventricular, *DLCO* diffusion capacity of the lung for carbon monoxide

**Fig. 2:**
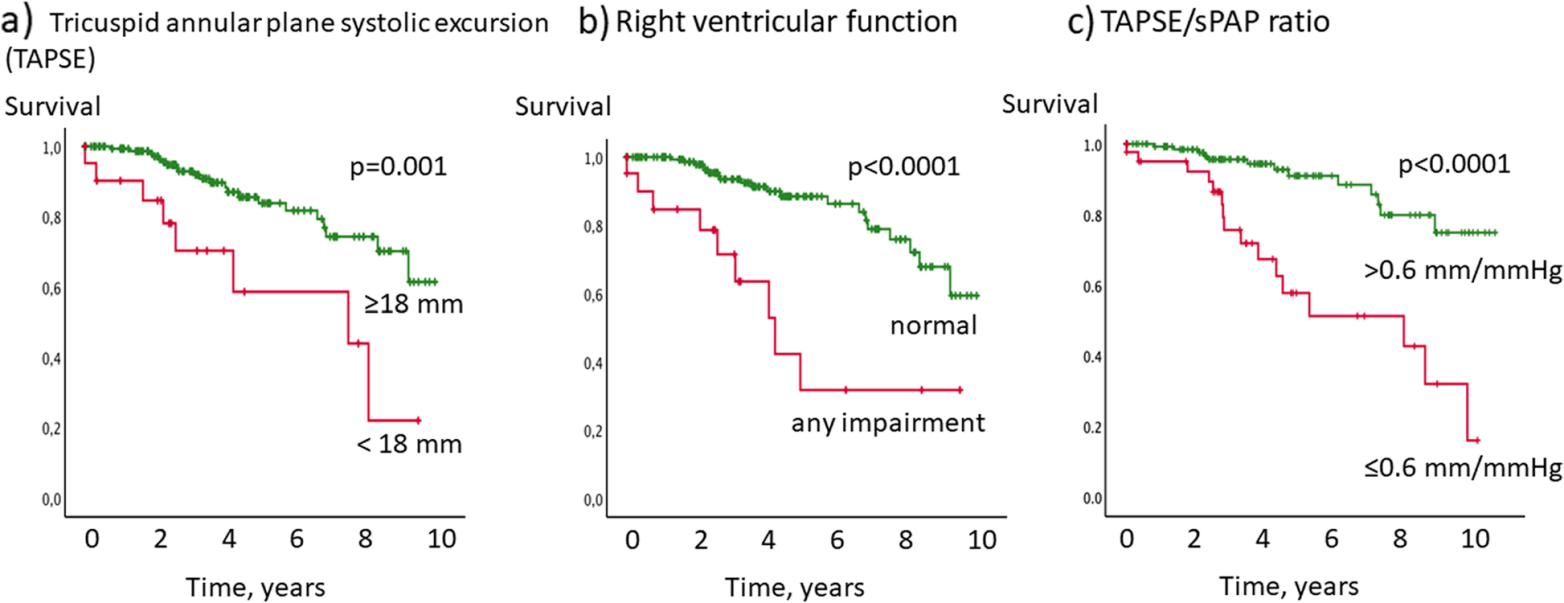
Kaplan-Meier survival analysis of echocardiographic right ventricular function. Patients with **a** tricuspid annular plane systolic excursion <18 mm assessed by echocardiography, **b** any impairment of RV function or with **c** TAPSE/sPAP ratio ≤0.6 mm/mmHg had significantly worse survival than patients with tricuspid annular plane systolic excursion ≥18 mm, normal RV function, or TAPSE/sPAP ratio >0.6 mm/mmHg

**Fig. 3:**
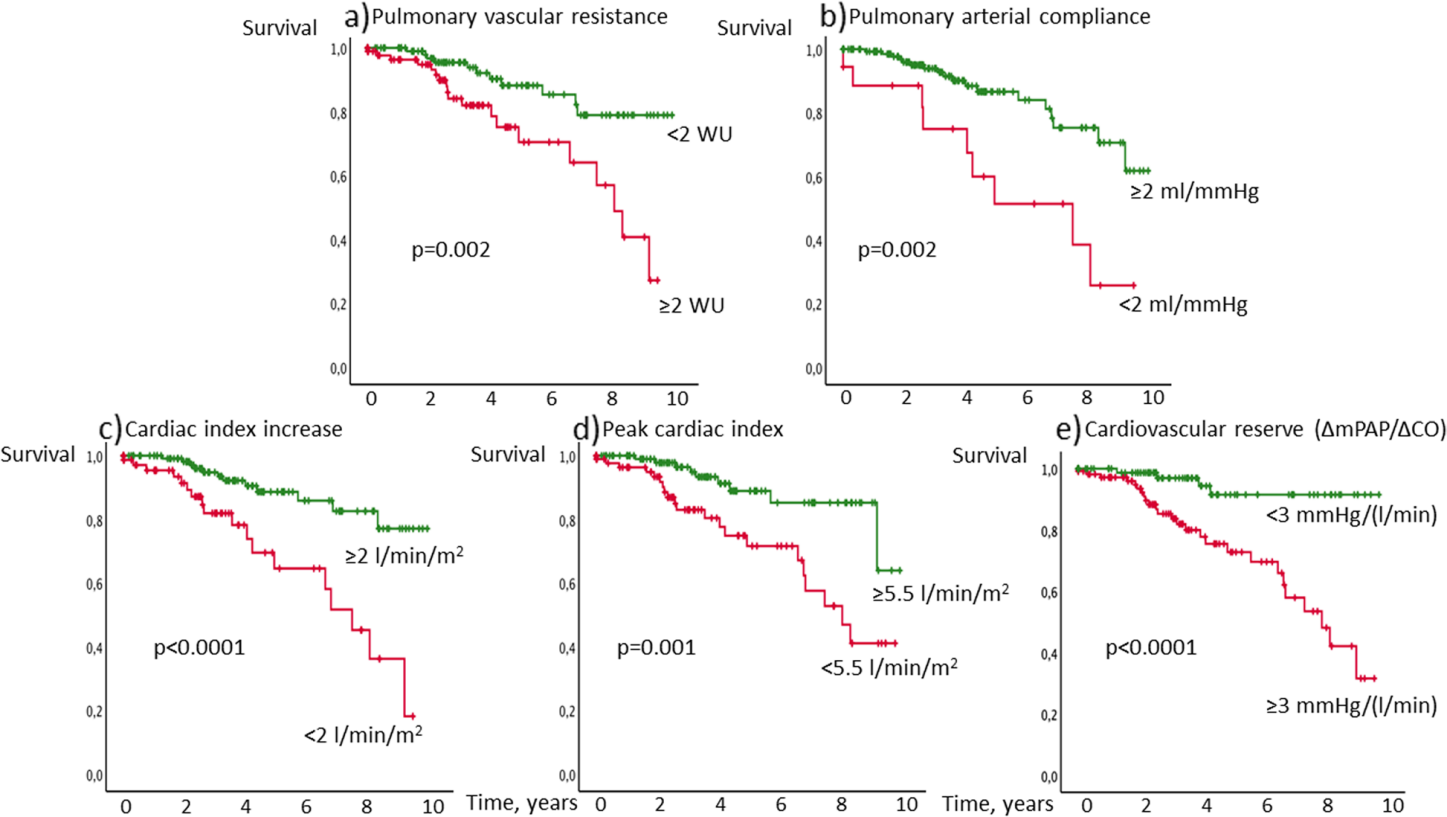
Kaplan-Meier analysis of invasively determined right ventricular function. Patients with **a** pulmonary vascular resistance ≥2 Wood Units, **b** pulmonary artery compliance <2 ml/mmHg, **c** cardiac index increase <2 l/min/m^2^, **d** peak cardiac index <5.5 l/min/m^2^ and/or **e** RV pulmonary vascular reserve (defined as the increase of mean pulmonary artery pressure/increase of cardiac output during exercise) ≥3 mmHg/(l/min) showed worse survival than SSc patients above the respective thresholds

Parameters of RV function significantly predicted survival in this patient cohort (Table 2, Figs. 2 and 3). Interstitial lung disease was significantly more often in patients with CI increase during exercise <2 l/min/m^2^ (45.6% vs. 29.1%, *p*=0.012). For other parameters, presence of ILD did not significantly differ between groups.

**Fig. 4:**
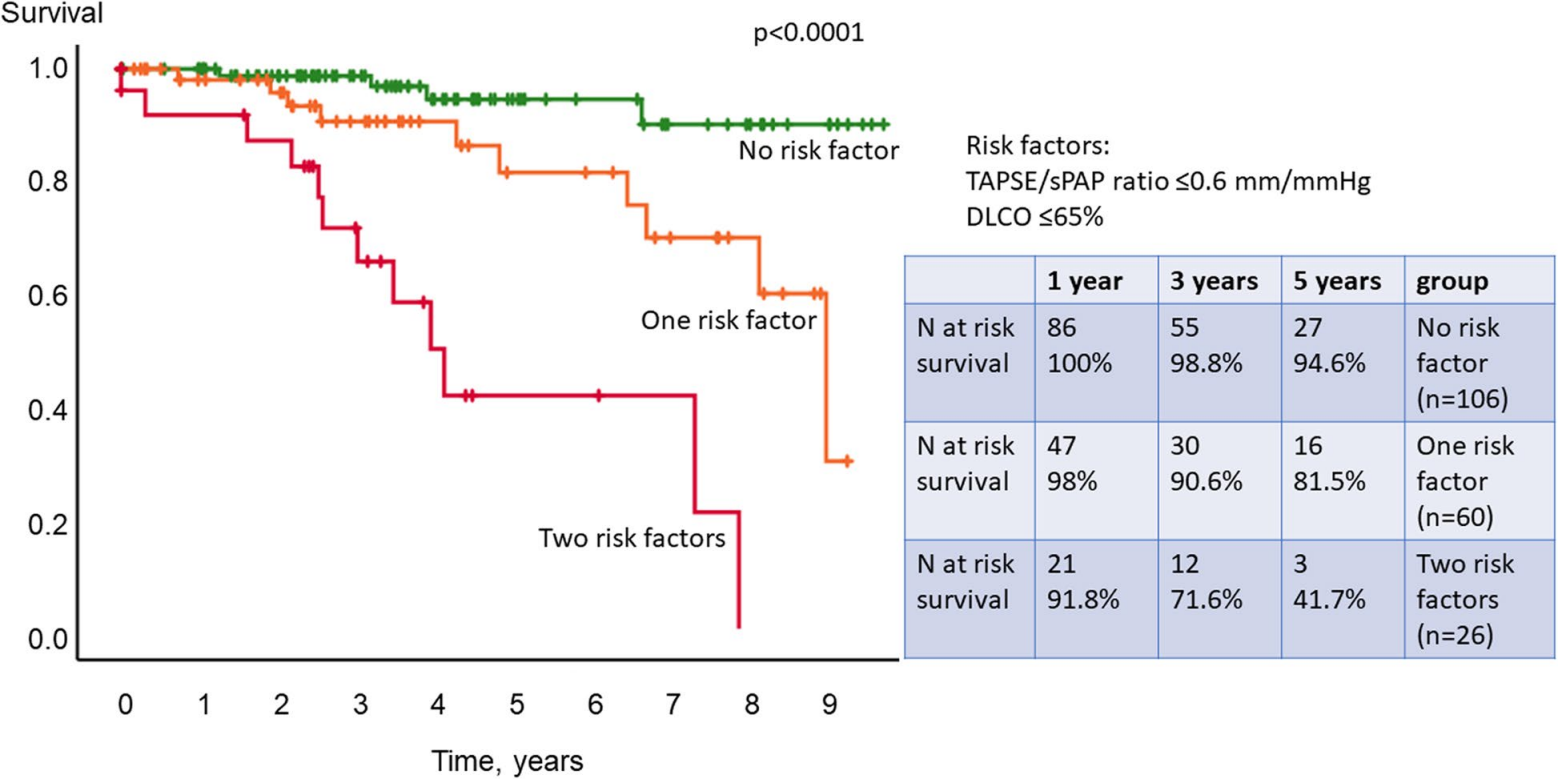
Kaplan-Meier analysis of multivariable risk set. Multivariable Cox regression analysis identified TAPSE/sPAP ratio ≤0.6 mm/mmHg and diffusion capacity for carbon monoxide of the lung (DLCO) ≤65% predicted as independent prognostic predictors of survival. Patients with none of these risk factors had significantly better survival than patients with one or two risk factors

In the *multivariable analysis*, TAPSE/sPAP ratio and DLCO ≤65% were identified as independent predictors of survival (Table 2). When combining these independent predictors, patients with no risk factor had significantly better survival than patients with 1 or 2 risk factors with 1-, 3- and 5-year survival rates of 100%, 98.8% and 94.6%, vs. 98%, 90.6% and 81.5% and 91.8%, 71.6% and 41.7% (Kaplan-Meier *p*<0.0001, Fig. 4). Prediction of survival remained significant when adjusted for age (*p*<0.0001). Independent prognostic predictors were confirmed by the sensitivity analysis leaving out patients with cancer as cause of death. In addition to the already identified parameters, type of SSc (*p*=0.004) and RV pulmonary vascular reserve (*p*=0.003) were identified as independent prognostic predictors in the sensitivity analysis.

When only patients with no signs of PVD at baseline (i.e. <21 mmHg mPAP or PVR <2 WU) were included in the multivariate analysis, CI increase during exercise <2 l/min/m^2^ was the only independent prognostic parameter for survival (*p*=0.028).

### Characterisation of patients in different risk groups

Patients with no risk factor (i.e. TAPE/sPAP ratio ≤0.6 mm/mmHg and DLCO ≤65% predicted) presented with significantly less haemodynamic impairment, better physical exercise capacity and better lung function (Table 3).

**Table 3:** Characteristics of different risk groups

	No risk factor (***n***=108)					One risk factor (***n***=66)						Two risk factors (***n***=26)						
	Mean ± SD or ***n*** and (%)			95% confidence interval	n*	Mean ± SD or ***n*** and (%)			95% confidence interval	n*	***p***-value (0 vs. 1 RF)	Mean ± SD or ***n*** and (%)			95% confidence interval	n*	***p***-value (0 vs. 2 RF)	***p***-value (1 vs. 2 RF)
**Characteristics**																		
Female sex, no [%]	90		83.3%			53		80.3%				23		88.5%				
Age [years]	55.5	±	12.1	53.2 to 57.9		59.4	±	13.9	56.0 to 62.8			60.9	±	13.5	55.5 to 66.4		0.002	0.20
Height [cm]	167.3	±	8.3	165.7 to 168.9		166.2	±	8.8	164.0 to 168.4			160.1	±	7.7	157.8 to 164.0			
Weight [kg]	72.8	±	15.4	69.8 to 75.7		70.6	±	16.7	66.5 to 74.7			66.3	±	15.9	59.8 to 72.7			
Type of systemic sclerosis																		
Limited cutaneous SSc [%]	80		74.1%			47		71.2%				19		73.1%				
Diffuse cutaneous SSc [%]	27		25%			17		25.8%				7		26.9%				
Early SSc [%]	1		0.9%			2		3%				0						
**Duration SSc [days]**	3398.6	±	3432.2	2737.6 to 4059.6	106	2646.8	±	2282.3	2081.3 to 3212.3	65		4829.9	±	4089.4	3178.2 to 6481.7			0.011
**WHO-FC, no [%]**																	Overall <0.0001	
I	27		25%			16		24.2%				2		7.7%				
II	60		55.6%			33		50.0%				6		23.1%				
III	21		19.4%			17		25.8%				16		61.5%				
IV	0					0						2		7.7%				
**Lung function**																		
Vital capacity max [l]	3.1	±	0.9	3.0 to 3.3	107	3.0	±	1.0	2.8 to 3.3	64		2.3	±	0.8			<0.001	0.004
Forced expiratory volume in one second [l]	2.5	±	0.8	3.4 to 2.7	107	2.4	±	0.8	2.2 to 2.6	65		1.9	±	0.6	1.6 to 2.1		0.001	0.010
Total lung capacity [l]	5.2	±	1.2	5.0 to 5.5	107	5.1	±	1.3	4.8 to 5.5	65		4.1	±	1.2	3.6 to 4.5		<0.001	0.001
Diffusion capacity of the lung for carbon monoxide SB [%]	66.4	±	16.4	63.3 to 69.5		49.8	±	13.1	46.5 to 53.0		<0.001	38.0	±	13.2	32.4 to 43.6	24	<0.001	0.004
Diffusion capacity of the lung for carbon monoxide/VA [%]	83.8	±	15.6	80.8 to 86.8		62.0	±	13.6	58.6 to 65.3		<0.001	48.0	±	12.0	43.2 to 52.8		<0.001	<0.001
**Laboratory**																		
N-terminal pro-brain natriuretic peptide [ng/l]	222.7	±	323.9	158.8 to 286.7	101	467.4	±	910.0	223.7 to 711.1	56		908.3	±	1291.9	335.5 to 1481.1	22	<0.001	0.048
C-reactive protein [mg/l]	6.6	±	10.0	4.7 to 8.5	107	5.2	±	4.7	4.1 to 6.4	64		10.0	±	9.6	6.1 to 13.8			
Glomerular filtration rate [ml/min/1.73m^2^]	87.8	±	24.4	83.0 to 92.5	103	87.1	±	29.6	79.2 to 94.9	57		76.3	±	25.1	65.1 to 87.4	22		
Creatinine [mg/dl]	0.78	±	0.18	0.75 to 0.82	107	0.87	±	0.55	0.73 to 1.0	64		1.33	±	2.38	0.36 to 2.29			0.023
**6-minute walking distance [meters]**	459.5	±	82.4	443.3 to 475.7	102	438.1	±	106.1	410.9 to 465.3	61		362.0	±	104.0	318.1 to 405.9	24	<0.001	0.003
**Echocardiography**																		
Right atrial area [cm^2^]	11.7	±	3.2	11.1 to 12.3	106	12.5	±	4.3	11.5 to 13.6	65		14.5	±	4.2	12.8 to 16.2	25		
Right ventricular area [cm^2^]	14.1	±	3.4	13.5 to 14.8	107	15.1	±	3.8	14.2 to 16.1			16.7	±	3.7	15.2 to 18.2			
Systolic pulmonary arterial pressure [mmHg]	26.3	±	5.5	25.3 to 27.3		32.1	±	12.2	29.1 to 35.1		0.001	55.4	±	17.3	48.5 to 62.4		<0.001	<0.001
Tricuspid annular plane systolic excursion [mm]	25.1	±	4.2	24.3 to 25.9		23.9	±	4.2	22.9 to 24.9			19.4	±	3.9	17.8 to 20.9		<0.001	<0.001
**Right heart catheter**																		
Mean pulmonary arterial pressure [mmHg]	17.3	±	5.1	16.4 to 18.3		20.2	±	6.8	18.5 to 21.9		0.019	34.9	±	10.7	30.5 to 39.2		<0.001	<0.001
Cardiac output [l/min]	5.6	±	1.5	5.3 to 5.9		5.3	±	1.3	5.0 to 5.7			5.1	±	1.2	4.6 to 5.6			
Pulmonary arterial wedge pressure [mmHg]	9.1	±	4.2	8.3 to 9.9		9.7	±	4.0	8.7 to 10.7			10.7	±	5.1	8.7 to 12.8			
Pulmonary vascular resistance [WU]	1.5	±	0.6	1.4 to 1.7		2.2	±	1.3	1.8 to 2.5		0.012	5.1	±	2.9	3.9 to 6.3		<0.001	<0.001
Pulmonary arterial compliance [ml/mmHg]	5.3	±	2.3	4.8 to 5.7	100	4.0	±	2.1	3.5 to 4.5	60	0.001	2.1	±	1.0	1.7 to 2.6	22	<0.001	0.001
RV pulmonary vascular reserve [mmHg/l/min]	3.1	±	2.0	2.8 to 3.5	107	6.1	±	15.2	2.4 to 9.8			9.6	±	7.6	6.5 to 12.6			
Cardiac index [l/min/m^2^]	3.1	±	0.8	3.0 to 3.3		3.1	±	0.8	3.1 to 2.9			3.0	±	0.5	2.8 to 3.2			
Cardiac index increase during exercise [l/min/m^2^]	3.3	±	1.6	2.9 to 3.6		2.7	±	1.5	2.4 to 3.1			1.5	±	0.8	1.2 to 1.8			
Peak cardiac index [l/min/m^2^]	6.4	±	1.7	6.1 to 6.7		5.8	±	1.7	5.4 to 6.2			4.5	±	1.0	4.1 to 4.9			
**Right ventricular pump function**																	Overall <0.0001	
Normal	105		98.1%			59		89.4%				15		57.7%				
Mild impairment	0					5		7.6%				4		15.4%				
Moderate impairment	0					0						4		15.4%				
Severe impairment	2		1.9%			2		3.0%				3		11.5%				

Only patients with assessment of both risk parameters were included in the analysis

In case of significant differences, *p*-values are provided

*RF* risk factor, *SB* single breath, *SSc* systemic sclerosis, *VA* alveolar volume, *WHO-FC* World Health Organization functional class

*In case of missing values, *n* is provided

Right heart size and pressure were significantly greater in patients with more risk factors. Though parameters of RV function were significantly more impaired in patients with one or two risk factors at baseline, CI at rest did not significantly differ between groups. The type of SSc and further laboratory parameters including C-reactive protein and glomerular filtration rate showed no difference between groups.

### Pulmonary vascular disease at follow-up

During follow-up, 86 patients who did not present with manifest PH at baseline had a follow-up RHC. Eleven developed manifest PH, 7 presented with mPAP ≥25 mmHg and PVR <3 WU and 10 had mPAP 21–24 mmHg and PVR ≥2 WU.

Sensitivity for prediction of PVD (mildly increased mPAP 21-24 mmHg and PVR ≥2 WU, increased mPAP ≥25 mmHg and PVR <3 WU or manifest PH) during follow-up for at least one risk factor was 75.0%, with a specificity (of not developing PVD during follow-up when presenting with no risk factor at baseline) of 69.0%. If two out of the three risk factors were present at baseline, the false-negative rate (not predicting PVD during follow-up) was 11.1%.

## Discussion

This is the first study illustrating that beside known prognostic predictors such as age, DLCO, presence of pulmonary fibrosis and elevated PVR, haemodynamic parameters of RV function at rest and during exercise are very important for risk stratification in patients with SSc, screened for PH. We identified reduced TAPSE/sPAP ratio and reduced RV pulmonary vascular reserve as new independent risk factors for survival. Furthermore, this study showed, that the newly identified multivariable risk set was able to predict the development of PVD during follow-up. In SSc patients who had no signs of PVD at baseline, RV output reserve, assessed by CI increase during exercise <2 l/min/m^2^ measured by RHC was the only independent prognostic parameter for survival. Thus, the results of this study suggest that RHC during exercise can give meaningful additional clinical information to identify patients, who need to be followed more closely or to receive an early treatment.

### Parameters of RV function and reserve

Impairment of TAPSE/sPAP ratio as parameter signalling beginning inability of RV to adopt to pressure elevation in pulmonary vasculature (RV-PA coupling) has been identified as the most important prognostic predictor in our cohort.

It has been previously shown that PAH patients with a TAPSE/sPAP ratio <0.31 mm/mmHg had a worse prognosis [48]. Almost all analysed parameters were significant predictors of survival in the univariable analysis. Known thresholds from the literature were feasible in our cohort of SSc patients including TAPSE/sPAP ratio ≤0.6 mm/mmHg, TAPSE of 18 mm [39], RV pulmonary vascular reserve of 3 ml/min/mmHg according to the current definition of exercise pulmonary hypertension [31].

For PAC, a threshold of 2 ml/mmHg was identified as prognostically relevant, predicting survival in our study cohort. The prognostic value of PAC has been investigated in several studies with thresholds for survival ranging between 1.26 and 2.5 ml/mmHg [49]. In an analysis of the PATENT and CHEST studies, a PAC ≥1.6 ml/mmHg was associated with better outcomes [50]. In patients with systemic lupus erythematosus-associated PAH, PAC <1.39 ml/mmHg was associated with impaired survival with a 3-fold higher risk of all-cause mortality and clinical worsening and was also the only independent predictor for both survival and clinical worsening [51].

As most studies on prognostic predictors in PH have investigated prediction models within Cox proportional hazard models using metric variables, thresholds for other parameters such as peak CI or CI increase during exercise have to be further investigated and validated. Recent data indicate that RV impairment and dysfunction already occur at 50% of peak oxygen uptake or even earlier [52]. Consequently, the timepoint of RV impairment might also provide information on RV function and reserve.

### Prognostic meaning of RV function and reserve in different entities

In our study, the main causes of death were P(A)H, pulmonary fibrosis and left heart disease. Both pulmonary fibrosis and left ventricular ejection fraction have been shown to be independently associated with RV free wall strain [53]. Parameters of the identified multivariable risk set included a known prognostic predictor (DLCO) and an echocardiographic parameter obtained at rest (TAPSE/sPAP ratio). In patients who did not present with signs of PVD at baseline, an invasively assessed parameter of RV function during exercise (CI increase) was identified as the only independent predictor for survival. It therefore covers a wide range of pulmonary, as well as vascular pathologies, which are important for different organ manifestations of SSc.

Exercise tests may unmask impaired RV function when the right ventricle at rest still looks normal and functions well. TAPSE/sPAP ratio has already been shown to be crucial for the identification of patients at risk for PH and has been used as tool to identify SSc patients in need of more extensive diagnostics by RHC [41]. The identification of RV output reserve as an independent risk factor in this study confirms the crucial role of exercise testing in patients with SSc to identify early stages of pulmonary hypertension which has been highlighted before [4, 13, 54–56].

### Clinical implications and future research

The identified multivariable risk set was not only able to predict survival, but also to identify patients at risk to develop PVD during follow-up. Whether parameters of RV function may be positively influenced by targeted treatment still remains to be investigated. Part of the pharmacotherapeutic effect may be attributed to indirect improvement of RV function by afterload reduction [57].

### Strengths and study limitations

This study enables important insights into different prognostic parameters and values representing RV function and reserve obtained from echocardiography and RHC from routine examinations. A major strength of the study is its real-world nature, which makes the study results more applicable to clinical practice. However, a prospective study including a complete set of RV function parameters and right heart catheterisation results also during follow-up to include positive and negative prediction would be desirable.

The reported clinical data were collected in a specialised PH centre, in which RHC during exercise is routinely performed in SSc patients presenting for PH screening. This quite “invasive” procedure is unusual in the management of SSc patients and needs to be revaluated prospectively. However, the described exercise parameters may help to detect a disease worsening at an earlier stage than the corresponding parameters at rest. Early prognostic markers need to be further investigated in future studies with a prospective design.

The study is limited by its retrospective design leading to inconsistent cohort sizes in the different analyses, because of missing values. Values for TDI TV s were missing in >15% of cases, for RV FAC and systolic PAP increase during exercise values were missing in >50% of cases. Furthermore, not all known parameters of RV function and reserve could be analysed (e.g. no strain at rest and during exercise, no magnetic resonance imaging assessment). However, the used parameters reflect the current clinical workup of patients with SSc and therefore offer real-life data of PH screening in SSc. Follow-up data of patients and changes of parameters over time were also not investigated in this study but would have been interesting with regard to screening and prognosis.

## Conclusions

This study highlights the importance of RV function and reserve assessments to predict prognosis and to identify patients at risk for development of PH. Exercise assessments may contribute to risk estimation. Therapeutic implications are still to be investigated.

## Data Availability

The datasets used and/or analysed during the current study were available in the patient files at Thoraxklinik Heidelberg gGmbH, Heidelberg.

## Abbreviations

6MWD: 6-min walking distance
APAH: Associated pulmonary arterial hypertension
CI: Cardiac index
CI: Cardiac output
DLCO: Diffusion capacity of carbon monoxide
ERS: European Respiratory Society
ESC: European Society of Cardiology
mPAP: Mean pulmonary arterial pressure
NT-proBNP: N-terminal pro-brain natriuretic peptide
PAC: Pulmonary arterial compliance
PAH: Pulmonary arterial hypertension
PAWP: Pulmonary arterial wedge pressure
PH: Pulmonary hypertension
PVD: Pulmonary vascular disease
PVR: Pulmonary vascular resistance
RHC: Right heart catheterisation
ROC: Receiver operating characteristic
RV: Right ventricular
RV-FAC: Right ventricular fractional area change
SD: Standard deviation
SSc: Systemic sclerosis
lcSSc: Limited cutaneous form of systemic sclerosis
dcSSc: Diffuse cutaneous form of systemic sclerosis
SSc-APAH: Systemic sclerosis-associated pulmonary arterial hypertension
TAPSE: Tricuspid annular plane systolic excursion
TDI: Tissue Doppler imaging
WHO-FC: World Health Organization functional class
WU: Wood Unit(s)
